# Treatment of Vestibulodynia with Submucosal Injections of IncobotulinumtoxinA into Targeted Painful Points: An Open-Label Exploratory Study

**DOI:** 10.3390/toxins15080476

**Published:** 2023-07-25

**Authors:** Paula Villa-Muñoz, Monica Albaladejo-Belmonte, Francisco J. Nohales-Alfonso, Jose Alberola-Rubio, Javier Garcia-Casado

**Affiliations:** 1 Servicio De Ginecología Y Obstetricia, Hospital Universitari I Politècnic La Fe, 46026 Valencia, Spain; vimupau@gmail.com (P.V.-M.); fnohalesa@gmail.com (F.J.N.-A.); 2 Centro De Investigación E Innovación En Bioingeniería (CI2B), Universitat Politècnica De València, 46022 Valencia, Spain; moalbel@ci2b.upv.es; 3 Sonda Devices S.L., 46004 Valencia, Spain; palberola.rubio@gmail.com

**Keywords:** vestibulodynia, vulvodynia, botulinum toxin A, sexual function, algometer, surface electromyography

## Abstract

The studies carried out to date on vulvodynia treatment with botulinum neurotoxin type A (BoNT/A) have followed generic injection protocols and reported contradictory outcomes on its effects. The aim of the present study was thus to propose a protocol for injecting BoNT/A into targeted painful points, to comprehensively assess the clinical effect of BoNT/A treatment and identify the risk/protective factors for successful treatment. Thirty-five vestibulodynia patients were treated with submucosal injections of incobotulinumtoxinA and assessed 8, 12 and 24 weeks after their treatment. Their clinical and pelvic statuses were assessed from self-reported questionnaires (Visual Analogue Scale (VAS), Female Sexual Function Index (FSFI), Marinoff’s Dyspareunia Scale (MDS), Hospital Anxiety and Depression Scale (HADS), Catastrophizing Scale (CS)), physical examinations and surface electromyography (sEMG). The patients reported a reduction in provoked vestibulodynia (<VAS, *p* < 0.01), improved sexual function (>FSFI, *p* < 0.01; <MDS, *p* = 0.01) and psychological status (<HADS, *p* < 0.01), and lower pelvic floor hyperactivity at rest (<sEMG amplitude, *p* = 0.01). Factors such as smoking, painful comorbidities, vulvar pain sensitivity and sexual function were significantly associated with successful treatment. The results indicate the beneficial effects of BoNT/A in treating vestibulodynia and reinforce the importance of adapting the treatment according to its clinical presentation and the patient’s medical background.

## 1. Introduction

According to the 2015 Consensus Terminology adopted by the International Society for the Study of Vulvovaginal Disease (ISSVD), the International Society for the Study of Women’s Sexual Health (ISSWSH) and the International Pelvic Pain Society (IPPS), vulvodynia is defined as vulvar pain that can be associated with different factors for at least 3 months with no identifiable cause [1]. It can be described according to its location (localized or generalized), its presentation (provoked or spontaneous), the onset of its symptoms (primary or secondary, i.e., whether or not after the first physical contact), and its temporal pattern (intermittent, persistent, constant, immediate or delayed) [1]. Provoked vestibulodynia is defined as vulvar pain in the vestibule caused by touching or sexual activity and with no clearly identifiable cause [2].

Unlike vulvar pain secondary to a specific disorder, such as recurrent candidiasis, postherpetic neuralgia or vulvovaginal atrophy, vulvodynia has no identifiable cause [3]. However, several factors have been associated with its development and perpetuation, for example, comorbidities and other painful syndromes; genetic polymorphisms associated with a higher sensitivity to pain; and hormonal, inflammatory, infectious, musculoskeletal, neurological, structural and psychosocial factors [1,2].

No epidemiological studies have been conducted to date on the prevalence of vulvodynia in the world population, although some authors reckon that it could affect 8–10% of women [4], with a higher incidence in young women under 30 years old and a similar incidence in sexually active women of different age groups. From an economic perspective, a non-probabilistic survey carried out in the USA calculated that the annual costs of vulvodynia were USD 31–72 billion, not including the costs associated with its psychological impact [5]. Vulvodynia also interferes with the patients’ and their partners’ sexual and mental health. Even though the role of anxiety and depression symptoms on the patient’s pain and sexuality is not known [4], some studies found that they show significantly lower desire, arousal and sexual satisfaction; a higher difficulty in achieving orgasm; a lower frequency of sexual intercourse; and a more negative attitude toward sexuality than healthy women, as well as negative changes in their sexual self-esteem and body image [4,6].

The diagnosis of vulvodynia usually involves a swab cotton test on the vulvar vestibule and an intravaginal digital examination of the pelvic floor muscles (PFMs). Both procedures rely on the clinician’s subjective perception and manual skills, and thus, tools such as algometers, which measure the pressure applied by the clinician on the vulvar vestibule in the swab cotton test [7], or techniques such as surface electromyography (sEMG), which provides objective information on the physiological and electrophysiological status of the PFMs [8], can be valuable for evaluating patients and used to monitor the treatment outcome. Self-reported clinical questionnaires are also widely used to assess the evolution of their physical, mental and social well-being after treatment [9].

There is a broad range of therapeutic options for vulvodynia (topical or oral medication, physical therapy, surgery, platelet-rich plasma, etcetera) with heterogeneous effectiveness among patients and are usually prescribed according to a stepwise plan [10,11]. Second-line treatments include injecting botulinum neurotoxin type A (BoNT/A) into the PFMs and/or vestibular mucosa. BoNT/A binds to peripheral nerve endings and inhibits acetylcholine release to the synaptic cleft, leading to transient muscle paralysis that lasts from 3–6 months when therapeutical doses are intramuscularly administered [12]. It also leads to releasing less neuropeptides that participate in pain signaling by blocking the neurotransmitter GABA and substance P. This translates into a reduction in the activation threshold of the nociceptors involved in afferent C-fibers, which show a higher number of nerve endings in vulvodynia patients. BoNT/A can, therefore, lead to a decrease in the peripheral and central sensitization associated with vulvodynia [3,13]. BoNT/A is used to treat multiple clinical disorders, such as muscle spasticity or cervical dystonia. Recent studies suggested administering it in the innervation zones of the muscle to enhance its therapeutic effect [14], and these can be detected with techniques such as Sihler’s staining technique [15] or high-density sEMG [16], as shown by recent research. While this may be true in neuromuscular disorders, current evidence does not support this technique in the case of vulvodynia and there is no standardized method of determining the injection sites.

While several studies obtained promising results from using BoNT/A for treating myofascial pelvic pain syndrome [17,18], those on its use in vulvodynia patients have reported diverse outcomes. The first of these were pilot studies carried out on a reduced number of patients with short follow-up periods and no randomization [19,20,21,22]. Even though they reported promising results, and thus, opened the door to the use of BoNT/A to treat patients with vestibulodynia, later studies with a more standardized methodology described contradictory outcomes [23,24,25]. They reported no differences between the patients and placebo groups for pain reduction after treatment, which could have been due to the low injected BoNT/A dose and the lack of a protocol to target painful points before performing the BoNT/A injections [23,24,25]. Furthermore, while pain perception, sexual function and quality of life were monitored in these studies, other relevant aspects that were shown to have a significant association with vestibulodynia pathophysiology, such as psychological well-being or PFM dysfunctions [3], were not followed up. Table 1 summarizes the characteristics of the studies that tested vestibulodynia treatment by injecting BoNT/A into the bulbocavernosus muscle. OnabotulinumtoxinA was injected into a single point on each side in all cases.

The aim of the present study was thus to assess the effect of BoNT/A injections into the vulvar vestibule on painful symptoms, sexual function, emotional functioning, and vulvodynia patients’ physical and electrophysiological PFM conditions. The secondary objectives were to implement a protocol for the evaluation and diagnosis of vestibulodynia to provide patients with the optimal treatment, to propose the criterion of clinically significant improvement after treatment and to identify any clinical characteristics that could influence its outcome.

## 2. Results

### 2.1. Sample Description

Table 2 shows the main general, obstetric, gynecological and pain-related characteristics of the 35 patients. Regarding their general medical history, their age was 41.9 ± 14.1 years old; their BMI was 22.4 ± 2.7 kg/m^2^; 22.8% of them smoked; and 8.5% had a history of sexual, physical and/or psychological abuse. In their obstetric background, 16 had previous pregnancies, 9 of whom (56.3%) had at least one vaginal delivery. Thirty patients (85.7%) had at least one painful comorbidity, among which cervical pain, lumbar pain, headaches and temporomandibular joint pain were the most common, as well as other comorbidities, such as allergies/intolerances. Regarding factors that could have precipitated vestibulodynia, 20% of the patients had suffered a spontaneous or iatrogenic vulvovaginal trauma, i.e., a second-degree perineal tear or an episiotomy; 17.1% had repeated vulvovaginal infections, among which 2.9% (one patient) had a history of genital herpes); and 34.3% had hormonal deficiencies.

As shown by their physical examination, all patients had superficial dyspareunia and 48.6% reported pain in the levator ani and/or internal obturator muscles of at least one of the two sides (left/right). Vestibulodynia was provoked in 60% of cases and mixed in the remaining 40% (14 patients), itching was the most prevalent symptom in patients with spontaneous vestibulodynia (13/14, 92.9%) and the mean number of years since vulvodynia onset was 6.9 ± 6.3. It should also be pointed out that three patients had previously been treated with BoNT/A injections in their vulvar vestibule, although they had been performed at least 1 year before enrolling in the study. The analyses were repeated excluding these patients, considering that previous treatment with BoNT/A could have introduced some bias in the assessments.

### 2.2. Evolution According to Clinical Questionnaires

**Figure 1:**
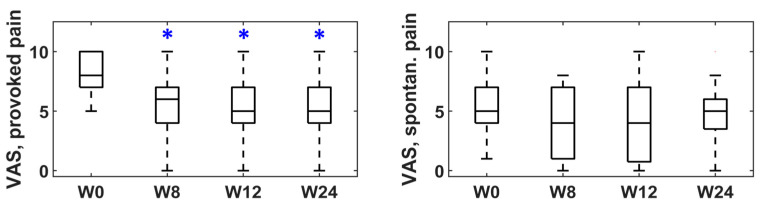
VAS scores for provoked and spontaneous vestibulodynia at baseline (week 0) and follow-up (weeks 8, 12 and 24). * statistically significant difference.

**Figure 2:**
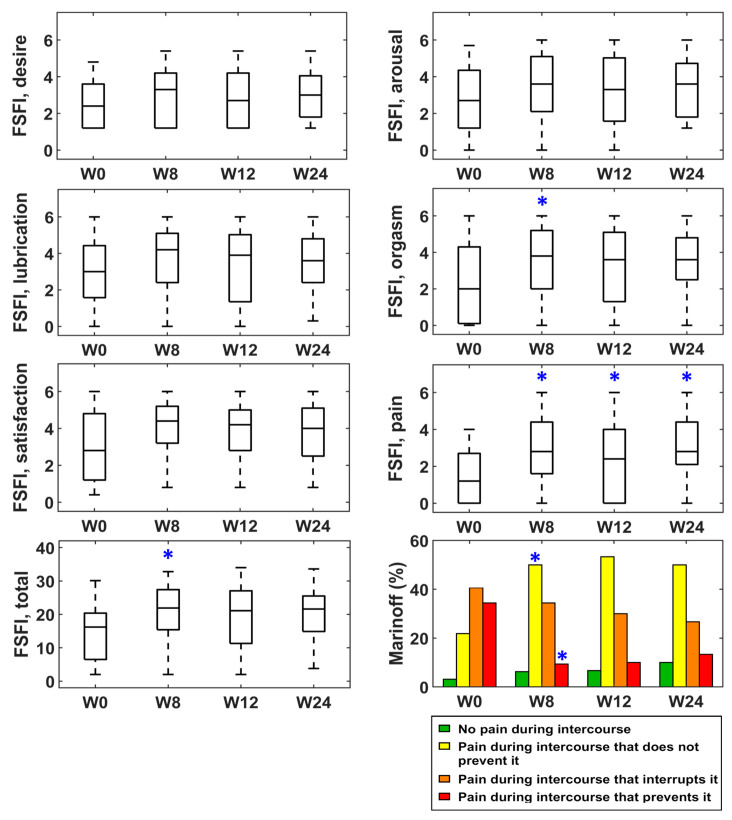
Scores of clinical questionnaires on sexual function at baseline (week 0) and follow-up (weeks 8, 12 and 24). * statistically significant difference.

**Figure 3:**
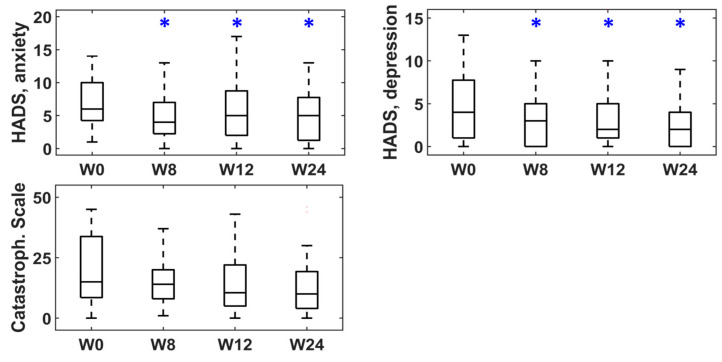
Scores of clinical questionnaires on psychological status at baseline (week 0) and follow-up (weeks 8, 12 and 24). * statistically significant difference.

Figure 1, Figure 2 and Figure 3 represent the box–whisker diagrams of the clinical test scores throughout the study. The follow-up visits that showed significantly different scores to those reported before treatment (week 0) are shown with an asterisk (primary outcome of the study). The mean ± SD (or frequency of occurrence of the categories) of the clinical tests and the *p*-values of the statistical comparisons performed are shown in the Supplementary Material (Table S1).

According to the VAS scores, the day-to-day pain reported by patients who suffered from provoked and spontaneous vestibulodynia was reduced after treatment, with statistically significant differences in all visits only in the former, as can be seen in Figure 1. 66.7%, 66.7% and 72.4% of the patients reported a reduction of at least 2 points in provoked VAS at weeks 8, 12 and 24.

Figure 2 shows that the total FSFI scores increased in all visits after treatment, although the *p*-values were only significant at week 8. FSFI scores in the pain domain were also higher and statistically significant differences were obtained for all follow-up visits. Marinoff’s Dyspareunia Scale also showed a statistically significant reduction in the percentage of patients that avoided intercourse because of pain at week 8 and an increase in those that only experienced a certain degree of discomfort that did not prevent intercourse.

In the patient’s psychological status, Figure 3 shows that the HAD-S questionnaire registered significantly lower anxiety and depression scores in the three post-treatment visits. The Catastrophizing Scale also showed lower scores after treatment, although the differences were not statistically significant in this case.

**Figure 4:**
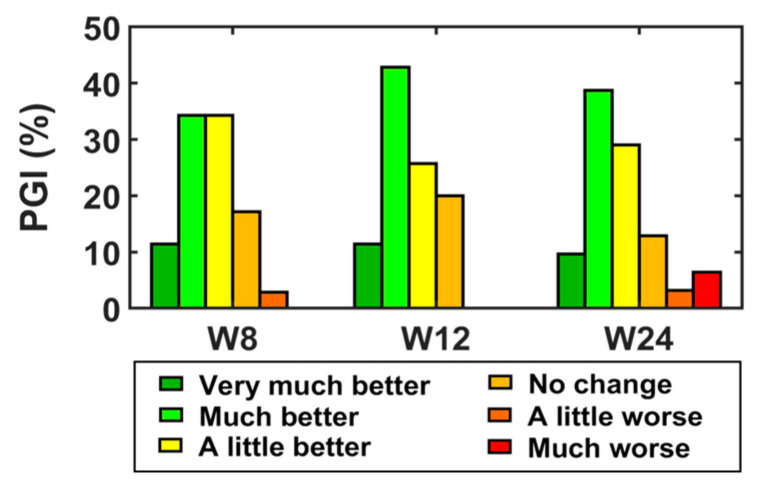
Frequency of PGI responses at weeks 8, 12 and 24.

Figure 4 summarizes the patients’ overall improvement in the three post-treatment visits according to their own perception (PGI-I). A clinically significant improvement (PGI: “much better” or “very much better”) was reported by 45.7% at week 8, 54.3% at week 12 and 46.9% at week 24. A total of 71.4% reported an improvement in at least one of the three follow-up visits, of which 40% reported an improvement in only one visit, 20% in two visits and 40% in all three post-treatment visits.

### 2.3. Evolution According to a Physical Examination and sEMG

**Figure 5:**
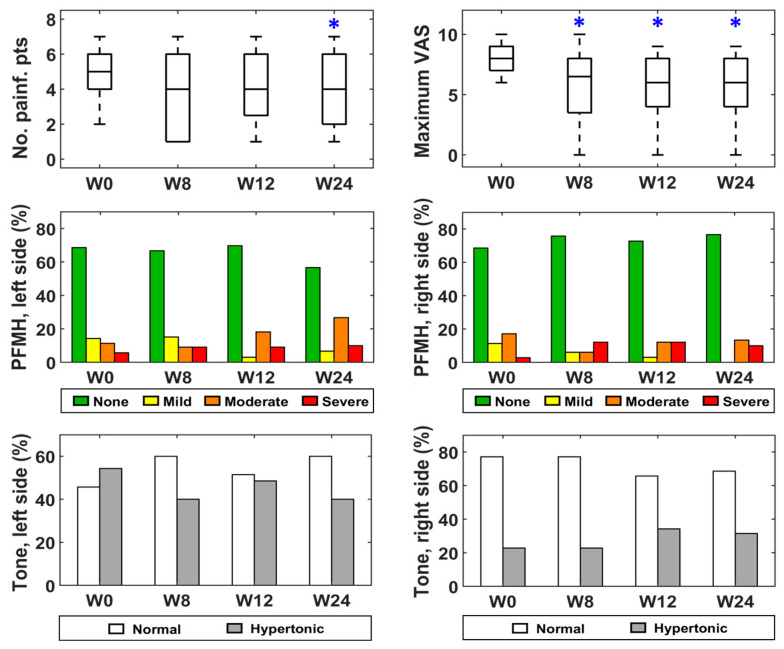
Physical examination at baseline (W0) and follow-up (W8, W12 and W24). * statistically significant difference.

**Figure 6:**
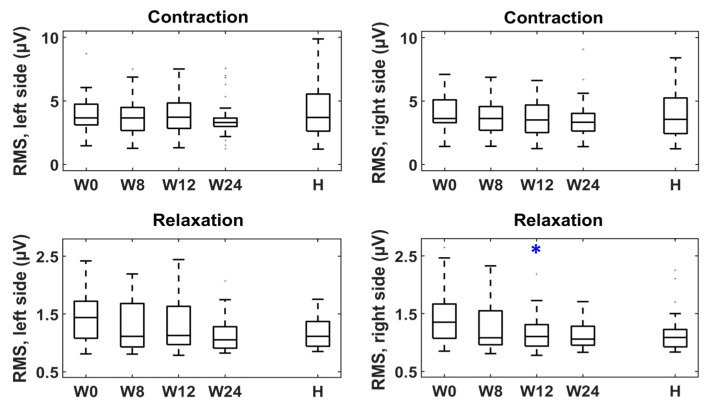
RMS of sEMG signals from patients (Ps) at baseline (W0) and follow-up (W8, W12 and W24) and from healthy women (Hs). * statistically significant difference (W0 vs. W8, W12 and W24).

Figure 5 shows the box–whisker graphs of the number of painful points in the vulvar vestibule and the maximum VAS reported in them during the cotton swab test, as well as the percentage of patients that showed hypertonic PFMs and every category of the PFMH score at weeks 0, 8, 12 and 24. Figure 6 gives RMS values of the patients’ sEMG signals recorded from both sides of the pelvis during muscle contractions and relaxations, plus others from healthy women. Statistically significant differences between the distributions at week 0 vs. weeks 8, 12 and 24 (primary outcome of the study) are highlighted with a blue asterisk in Figure 5 and Figure 6.

It can be seen in Figure 5 that there were fewer painful points after treatment, reaching a minimum at week 24. It also gives the maximum VAS scores, which reached their minimum at week 8. The patients’ RMS values during relaxation also decreased in post-treatment visits, although statistically significant differences were only obtained on the left side at week 8. Furthermore, their medians showed a higher trend of values than those of healthy women at week 0, and of more similar values in subsequent weeks, although statistically significant differences were not obtained for any visit when Bonferroni corrections were used (Figure 6). On the other hand, the percentage of patients that showed hypertonic deep PFMs did not change in the follow-up visits, nor did the PFMH score distribution (Figure 5).

### 2.4. Adverse Events

Only two patients reported adverse events after the BoNT/A injections, which were stress urinary incontinence in one case and difficulties in achieving climax in the other. Their onset, duration and severity are summarized in Table 3, which shows that both were mild and transient adverse events that were reported by the patients in the follow-up visit scheduled at week 8 but had disappeared at weeks 12 and 24.

### 2.5. Analysis of Risk Factors

Regarding the secondary outcome of the study, the “responder” and “non-responder” groups contained 21 (60%) and 14 (40%) patients, respectively. Table 4 shows the patients’ baseline clinical characteristics that were significantly associated with either a risk factor for a non-clinically significant improvement after the BoNT/A treatment or a protective factor against it. It can be seen that smoking, the presence of any of the associated syndromes listed in the sensitization clinical questionnaire (migraine or tension headaches, fibromyalgia, chronic fatigue syndrome, etc.) and higher FSFI orgasm scores were associated with risk factors, while the perception of pain at the 3 o’clock position in the vulvar vestibule and higher maximum VAS scores in the cotton swab test were associated with protective factors.

### 2.6. Sensitivity Analysis: Exclusion of the Patients Previously Treated with BoNT/A Injections into the Vestibule

The same statistically significant differences between the patients’ clinical characteristics at week 0 vs. weeks 8, 12 and 24 (primary outcome) were obtained when the three patients previously treated with BoNT/A injections into their vestibule were excluded from the analysis. The only exceptions were the FSFI score in the orgasm domain, which also increased at week 8, but not statistically significantly when the significance level was corrected (*p*-value = 0.008, *α* = 0.05/21), and the Marinoff’s Dyspareunia Scale (*p*-value = 0.03, *α* = 0.05/3). The analysis of risk factors (secondary outcome) also provided similar results to those shown in Table 4, although “Vestibular pain at the 3 o’clock position” was no longer significant (OR [95% CI] = 0.18 [0.03–1.18], *p*-value = 0.06) after excluding the three patients.

## 3. Discussion

### 3.1. Patients’ Evolution after Treatment: Clinical Questionnaires

One of the main objectives of the present study was to assess whether patients’ day-to-day pain diminished after BoNT/A injections according to the VAS metric, which has been widely used in previous studies on the use of the toxin for treating vulvodynia [20,23,24,25,26,27]. Our results showed a statistically significant reduction in VAS scores given by patients to provoked vestibulodynia, but not of those given to spontaneous vestibulodynia. This could be explained by an underlying undiagnosed neuropathy if the Nantes criteria were not strictly met in the previous visit [28] and/or the reduced sample size and margin for improvement, as only 40% of the patients showed spontaneous vestibulodynia, which was mild in many cases. Given that there may be differences in the pathological mechanisms underlying provoked and spontaneous vestibulodynia, such as a higher central nervous system involvement/dysregulation in the latter [29], our results could also imply that the benefit from BoNT/A treatment may be limited to provoked pain and not spontaneous pain. The reductions in provoked pain found in the present study are similar to those reported by previous studies in the field. In the present study, the patients showed a severe mean VAS (8.09) at baseline, which was reduced and became moderate in the follow-up visits, as in the studies by Petersen et al. [23] and Haraldson et al. [25]. As in Petersen et al. [23], around 50% of the patients reported a drop in the VAS scores of at least 2 points in all the follow-up visits.

Similar to previous studies on the effect of BoNT/A for treating provoked vestibulodynia [23,27], the FSFI scores showed significantly higher values at follow-up, implying an improvement in patients’ sexual function. As in the study by Diomande et al. [24], this improvement was also assessed using Marinoff’s Dyspareunia Scale, as it provides additional information on the patients’ painful sensations during intercourse. According to both questionnaires, full sexual function recovery was only observed in a few cases in our sample, probably because vestibulodynia patients have more severe sexual function impairment than in other chronic vulvar conditions [26].

It is known that the patient’s psychophysiological status can influence the effect of drugs in drug trials [3] and some studies suggested that psychological discomfort is a consequence rather than the cause of vulvodynia [30]. For this reason, unlike previous studies, the patient’s emotional functioning was also assessed in the present study before and after treatment by the catastrophizing scale and HADS. As with the other clinical aspects evaluated, the changes reported in HADS scores indicated an improvement in some aspects of their emotional functioning after treatment with BoNT/A.

### 3.2. Patients’ Evolution after Treatment: Physical and Electrophysiological Exams

In addition to the patients’ day-to-day pain related to provoked vestibulodynia, their pain perception during the cotton swab test was also assessed, as dyspareunia is not necessarily related to pain with physical examination [9]. This, indeed, was observed in the patients of the present study at the follow-up: some of them reported an improvement in provoked pain during both intercourse and the cotton swab test, while others only reported pain in one of them. Even so, the highest VAS scores during the cotton swab test were significantly lower in post-treatment visits, as well as their mean values (results not shown), which could not be demonstrated in the only previous study that also monitored this outcome [24]. As for the sEMG findings, the patients’ signal amplitude during PFM relaxation showed a trend of higher values than that of their healthy counterparts at baseline but became similar at follow-up. Although this trend was not statistically significant, it agrees with the findings of a previous study that treated patients with vestibulodynia under a physical therapy program [31].

### 3.3. Patients’ Evolution after Treatment: Adverse Events

Adverse events associated with BoNT/A’s effect were scarce, temporary and self-limited, probably due to the maximum dose being restricted to 200 units Only one patient reported mild stress urinary incontinence and another reported difficulties in achieving climax at week 8, but not at weeks 12 and 24. While a previous study also reported mild urine leakage after BoNT/A injections [25], this was the first study reporting difficulties in achieving climax as an adverse event of the treatment. As higher PFM strength is associated with better sexual function and particularly with a greater facility to achieve climax [32], this adverse event could be explained by BoNT/A’s chemical denervation of motor units in the muscle, which thus became unable to contract and develop strength. As both adverse events were mild and transient, this study adds to previous findings that confirmed BoNT/A injected in limited doses can be safely used to treat vestibulodynia [33].

### 3.4. Response to BoNT/A: Definition of Clinically Significant Improvement

The PGI-I scale was used to identify the patients that experienced a clinically significant improvement after the BoNT/A treatment. Our group carried out a similar patient classification and assessment of risk factors in deep dyspareunia treatment with BoNT/A [18]. However, the criterion used to classify patients as “responders” and “non-responders” in that study relied on VAS changes from baseline to follow-up visits. The reason why the PGI-I scale was used in the present study was that it assesses improvement from an overall perspective that implicitly includes psychological well-being and other aspects related to the patient’s quality of life, and not only the perception of pain, as occurs with VAS. This was also the reason why FSFI [23] or cotton-swab-provoked VAS [24] were not used either.

Twenty-five patients showed a clinically significant overall improvement in at least one follow-up visit, of which four patients reported this improvement only at week 24. Given what is known about the onset and the maximum effect of the toxin [34], the improvement of these four patients would not be associated with its effect but with other external factors; therefore, the PGI-I scores at week 24 were not considered to group the patients as BoNT/A responders and non-responders.

### 3.5. Response to BoNT/A: Risk and Protective Factors

The results obtained from the analysis of the risk factors agreed with those reported in previous studies [23,24] since age, duration of symptoms, day-to-day VAS and total FSFI scores at baseline were not associated with a risk or protective factor against a clinically insignificant improvement after treatment. However, the present study assessed a higher number of variables from the patient’s medical history, some of which showed up as significant factors. According to our results, smoking, painful comorbidities and high scores in the FSFI orgasm domain before treatment were associated with a higher risk of no improvement after the BoNT/A treatment. Smoking may lead to abnormal pain-processing mechanisms [35] and is associated with vulvovaginal atrophy [36], which is a cause of vulvar pain. BoNT/A treatment would not have any effect on this cause of pain, thus explaining why this habit showed up as a risk factor in our study. As for painful comorbidities (headaches, fibromyalgia, painful bladder syndrome), it is known that almost half of vulvodynia patients suffer from other types of chronic pain that have common altered neuronal, immune and endocrine mechanisms that lead to dysfunctional sensorial and pain processing [2].

Higher values of the FSFI orgasm domain could be associated with an underlying injury or compression of the pudendal nerve, as these affections can lead to higher excitability, and thus, affect orgasm during intercourse [37]. Although a diagnosis of pudendal neuralgia was ruled out in the patients during their physical examination, there could have been some subclinical involvement of this nerve in the etiology of vulvodynia, especially in the non-responders. This would explain why higher values in the FSFI orgasm domain were associated with a risk factor for a worse response to treatment.

On the other hand, the factors associated with a clinically significant improvement after the BoNT/A treatment included higher maximum VAS scores in the cotton swab test and pain in the 3 o’clock position. The first outcome could have been due to a larger margin of improvement caused by higher vulvar pain sensitivity at baseline. As for the second protective factor, it could be related to the fact that the 3 o’clock position is on the left side of the vestibule and the prevalence of PFM hypertonicity was higher on this side than the other in our patients, although further efforts should be made to determine the reason for this result.

### 3.6. Strengths and Limitations

One of the study’s strengths was the use of maximum BoNT/A doses and the standard selection of painful vestibular points to perform the injections according to a protocol that relied on an algometer. The limited effectiveness attributed to BoNT/A in previous studies could have been related to these two aspects since they administered lower BoNT/A doses (up to 100 units) and systematically infiltrated the 5 and 7 o’clock points of the bulbocavernosus or vestibular mucosa [20,23,24,25,26,27]. While these positions are relevant since the pudendal nerve enters the vulva posteriorly [24], identifying painful areas is one of the most important aspects when planning treatment [38]. A second strength was the meticulous method of recruiting patients. Most of the medical conditions excluded were identifiable causes of gynecologic pain that commonly overlap with vulvodynia but on which BoNT/A is not anticipated to have any therapeutic effect, implying that they would have masked changes in vulvodynia symptoms after treatment. Patients who showed any of these conditions were previously treated with more suitable approaches (e.g., hormone replacement therapy), and thus, they were included in the study only if these conditions resolved and the vulvar pain could no longer be related to any identifiable cause. A third strength was the thorough follow-up carried out 8, 12 and 24 weeks after the BoNT/A injection. Unlike previous studies [20,23,24,25,26,27], the outcomes monitored in post-treatment visits were not only related to the patients’ pain perception, sexual function and vulvar sensitivity but also to their emotional functioning and PFM physical and electrophysiological status, which brought a more comprehensive perspective of the patient’s evolution.

The study’s main limitation was that it lacked a placebo group, and thus, its design had to be non-randomized, which prevented it from making causal inferences to determine BoNT/A’s true effect in terms of pain reduction; and non-masked, which could have introduced some bias in the interpretation of the results. Previous studies that included a control group reported an improvement not only in those treated with BoNT/A but also in those that received injections of a saline solution [23,24,25], indicating that the positive outcomes obtained in this study could be justified by the puncture effect rather than the toxin itself. Further efforts should thus be made to recruit a control group and test BoNT/A’s placebo effect. Other aspects that should be evaluated in more depth include the effectiveness of repeated injections, as this was only evaluated by Diomande et al. in an exploratory analysis [24], plus the influence of injecting the toxin into the bulbocavernosus vs. vestibular mucosa.

The participants in the study were recruited at a specific pain consultation at a specialized unit, which could also raise concerns about possible selection bias. However, the patients evaluated by this unit are referred to it after they have been examined at a general or emergency gynecology consultation and if their painful symptoms persist after treatment with conventional medication. Rather than being a limitation of the study, we believe that this was one of its strengths, as the expertise of the clinician who carried out the pain consultation allowed him to effectively discern whether vulvar pain was associated with an identifiable factor, in which case medication other than BoNT/A should be prescribed. On the other hand, patients were from the same geographic region, and thus, the results may not be representative of the broader population. Furthermore, while the number of follow-up visits in the study and their scheduled times allowed us to confirm that the onset of vulvodynia relief and its duration varied widely, a longer follow-up might have helped to determine the treatment’s sustained effects or potential complications and this should also be taken into account in future studies.

## 4. Conclusions

As there is still much to be explored in the field of vulvodynia, studies that provide further insights into its diagnosis and treatment and avoid neglecting these patients, as they have been for years, are of vital importance. While they have diverse treatment options, the most potentially effective should be chosen after a thorough anamnesis and examination of the patient.

The present study was an open-label exploratory study that proposed a standardized protocol to treat vestibulodynia with incobotulinumtoxinA injections at a maximum dose of 200 units It combined subjective and objective tools to target painful points in the vulvar vestibule and reported the clinical evolution of these patients that were treated with the toxin and subsequently monitored for up to 24 weeks. Our results showed a reduction in vestibulodynia-associated pain after incobotulinumtoxinA submucosal injections, as well as an improvement in the patients’ sexual function, psychological status, vulvar pain sensitivity and PFM hyperactivity. Some aspects related to the patient’s medical background, sexual function and physical examination, such as smoking, painful comorbidities, orgasm during intercourse and the location and intensity of vestibular pain, were found to be associated with a clinically significant improvement after the BoNT/A treatment, thus reinforcing the widespread opinion that vestibulodynia treatment must be planned according to the individual patient’s clinical profile. In spite of these promising findings, further efforts should be made to assess BoNTA/A’s true clinical effect by including a placebo group in the study.

## 5. Materials and Methods

### 5.1. Study Overview

The present study, which was conducted at the Hospital Universitari i Politècnic La Fe (Valencia, Spain), was a prospective, minimally invasive, non-masked and non-randomized study entitled “Study of the pelvic floor muscles after botulinum toxin infiltration for vulvodynia treatment” (Spanish Agency of Medicines and Medical Devices code: JAR-TOX-2019-01). The study adhered to the Helsinki Declaration and was approved by the institutional ethics board. Patients were informed of the nature of the study, treatment procedure and sEMG recording protocol, and they provided their informed consent at least 24 h before the BoNT/A injection. The vulvodynia patients were recruited in a specific pain consultation at the Pelvic Floor Unit in the Obstetrics and Gynecology Service of the hospital by a specialized gynecologist. The exclusion criteria were as follows: under 18 years old, active pelvic/vulvovaginal infection, untreated vulvar atrophy, known dermatological lesion, pudendal neuropathy with signs of entrapment, musculoskeletal/genitourinary conditions, previous genital surgery or trauma that could interfere with the study, malignant or psychiatric disease, drug allergies, treatment with another drug under study in the 30 days prior to the start of the present study, contraindications against BoNT/A administration (included in the datasheet), pregnancy and breastfeeding.

The study was structured as in the study by Tarazona-Motes et al. [18] and consisted of a previous visit; a visit in which treatment was performed (week 0); and three follow-up visits scheduled for 8, 12 and 24 weeks after BoNT/A injection (weeks 8, 12 and 24, respectively). Table 5 shows the procedures and activities carried out at each visit with a cross (X). Thirty-eight patients were recruited, of which two were lost to follow-up before the first follow-up visit and one was excluded for showing a diagnosis other than vulvodynia as the main chronic pelvic pain symptom. Of the 35 patients in the final study sample, three dropped out of the project at week 24.

### 5.2. Medical History

The data pertaining to the following categories were collected from the patient’s medical histories: social and demographic information (age, educational qualifications, occupation), anthropometric (body mass index), medical background (disease or surgery), obstetric and gynecological background (vaginal deliveries, cesarean sections, second- or third-degree perineal tears, menopause, fibroids, polyps, etc.), urogynecological symptomatology (dysmenorrhoea, ovulatory pain and urinary incontinence), musculoskeletal symptomatology (diarrhea, abdominal pain, etc.) and other concurrent treatments.

Anamnesis was focused on pelvic pain dysfunction and painful symptoms (vulvodynia, years since pain onset, suspicion of comorbidities, pudendal neuropathy and myofascial pelvic pain syndrome) and sensitization in pelvic pain was identified according to the clinical questionnaire designed by Levesque et al. [39]. Patients were also asked about sexual, physical and psychological abuse and treatments received.

### 5.3. Monitoring Clinical Status

The patient’s clinical status was assessed according to clinical questionnaires. Day-to-day pain associated with provoked and spontaneous vulvodynia was independently rated using the Visual Analogue Scale (VAS) [7], sexual function was assessed according to the Female Sexual Function Index (FSFI) [40] and Marinoff’s Dyspareunia Scale [41], and the patient’s emotional functioning was evaluated using the Hospital Anxiety and Depression Scale (HADS) [42] and the Catastrophizing Scale [43]. The patient also rated from a global perspective the clinical improvement experienced after the BoNT/A treatment using the Patient Global Impression of Improvement (PGI-I) rated on a 7-point scale: “very much better”, “much better”, “a little better”, “no change”, “a little worse”, “much worse” and “very much worse” [44].

### 5.4. Monitoring Pelvic Physical Status

The physical examination was focused on the assessment of the pudendal nerve, the deep PFMs and the vulva. The absence of genital prolapse had been confirmed in the previous visit.

Pudendal nerve assessment consisted of detecting signs of pudendal neuropathy, such as painful ischiatic spines, perineal surface tenderness, bulbocavernosus reflex and the painful “skin rolling test” [45].

The deep PFM examination included (1) evaluation of their tone via digital palpation performed with one finger inserted in the vagina and (2) the rating of the pain experienced by the patients during palpation according to the pelvic floor muscle hyperalgesia (PFMH) scoring system [46]. Both aspects were assessed separately on the left and right PFM sides.

The vulvar assessment consisted of evaluating trophism and tenderness. For the latter, the patient was asked to rate the pain experienced (according to VAS) in the {1,3,5,6,7,9,11} clock positions of the vulvar vestibule when a pressure of 0.2–0.4 kgfwas applied by a cotton swab inserted in a digital algometer (Wagner FPIX™, WAGNER INSTRUMENTS, Greenwich, CT, USA). Painful points related to provoked vestibulodynia were thus identified as those at which the patient reported moderate or severe pain (VAS ≥ 4 [47]) with the exerted pressure.

### 5.5. Monitoring Pelvic Floor Muscle Electrical Activity

The PFM electrical activity was monitored using sEMG and detected with six Ag/AgCl self-adhesive electrodes (Red Dot 2660-5, 3M, St. Paul, MN, USA), with four attached to the labia majora and the two others to the right (reference electrode) and left (ground electrode) ischial tuberosity. An abrasive gel (Nuprep 114 g, Weaver and Company, Aurora, CO, USA) was previously used to gently exfoliate the skin to reduce the skin–electrode impedance.

One bipolar signal was obtained from each PFM side (left, right) as the difference between the electrical activity detected by the upper and lower electrodes attached to that side. The signals were acquired by a multipurpose amplifier (Grass 15LT + 4 Grass 15A94, Grass Instruments, West Warwick, RI, USA) with a gain of 20,000, a band-pass bandwidth of [3, 1000] Hz and a sampling rate of 10 kHz. Recordings were performed while the patient carried out a protocol of five 5 s maximum voluntary contractions of the PFMs, as if holding urine, interspaced by 10 s rest periods. To reduce crosstalk in the sEMG recordings, the clinician checked that the patients did not contract other nearby muscles, such as the abdominal, gluteal or lower limb muscles, while contracting the PFMs. The first contraction was executed after a 1 min resting period during which the patient was asked not to contract her PFMs.

The bipolar signals were filtered and five contraction segments and one with resting activity were manually annotated in each (see further details in [48]). The root mean square (RMS) of the annotated signal segments was computed as an estimator of the amplitude of PFM tonic and voluntary activity and compared with the RMS of sEMG signals from healthy women recorded using the same electrode arrangement and voluntary contraction protocol [49].

### 5.6. BoNT/A Administration

A local topical anesthetic (EMLA, Aspen Pharmacare España S.L., Barcelona, Spain) was applied to the vulvar vestibule before injecting BoNT/A to limit the pain associated with the injection and was then removed with a gauze dressing soaked in chlorhexidine. IncobotulinumtoxinA (Xeomin^®^, Merz Pharmaceuticals GmbH, Frankfurt, Germany) was then injected in the painful positions of the vulvar vestibule identified by the cotton swab test with the aid of the algometer during the physical examination for provoked vestibulodynia. A dose of BoNT/A of at least 25 units and at most 33 units was administered at each painful point. The exact dose injected at individual points was selected to ensure that the maximum total dose did not exceed 200 units to avoid adverse events, such as severe urinary incontinence, and to prevent a waste of part of the 100-unit vial content when possible, which was diluted in 1 mL of lidocaine at 2% and 9 mL saline. The maximum dose limit was set to 200 units according to the clinician’s clinical expertise and the highest dose used in previous studies [24].

### 5.7. Data Analysis

The clinical characteristics collected from the patients in the previous visit were first summarized according to their mean ± standard deviation (SD) or the frequency of occurrence (%) of their categories in the sample, depending on whether they were numerical or categorical, respectively.

Statistically significant differences between the scores of the clinical questionnaires and the physical and electrophysiological status of the pelvic floor at week 0 vs. weeks 8, 12 and 24 were the primary outcomes of the study. Day-to-day provoked and spontaneous VAS, FSFI, HADS and Catastrophizing Scale scores in the two visits were compared, as well as the number of painful vulvar points and their mean VAS during the cotton swab test and the sEMG signal amplitude in these visits, using the paired-sample t-test or the Wilcoxon signed-rank test, depending on the normality of the data, which was assessed using a Kolmogorov–Smirnov test. Statistically significant differences between the sEMG signal amplitude of the patients at weeks 0, 8, 12 and 24 vs. healthy women were evaluated according to the two-sample t-test (normal data) or the Mann–Whitney U test (non-normal data). The frequency of the four Marinoff Dyspareunia Scale levels in the two assessed visits was compared using the Stuart–Maxwell test and, in the cases with a statistically significant difference, a post hoc analysis based on McNemar’s test identified the option whose frequency was significantly different in the two visits. The Stuart–Maxwell test and a subsequent post hoc analysis were also performed to assess statistically significant differences between both the tone and the PFMH score of the deep pelvic floor muscles at week 0 vs. weeks 8, 12 and 24. A questionnaire/exam was considered to show a statistically significant clinical change after treatment if the scores of any of its sections were significantly different from baseline at any follow-up visit. To avoid increasing the probability of making a type I error, the significance level of the statistical tests in that questionnaire/exam was set to *α*/*m* (*α*: 0.05, *m*: number of sections in the questionnaire or exam x number of follow-up visits), according to the Bonferroni correction.

The secondary outcome of the study was the identification of clinical characteristics that may influence the outcome of BoNT/A treatment through a responder analysis, which was performed according to the patient’s overall clinical improvement after it. The criterion used to determine whether they had experienced a clinically significant improvement was based on their PGI-I scores, such that the “responders” group included those who indicated that their clinical condition was much or very much better [50,51,52] in at least one of the two first follow-up visits, while the “non-responder” group included the remaining patients (the criterion selected is discussed in Section 4 of the manuscript). A univariate binary logistic regression analysis was thus performed to assess whether the patients’ baseline clinical characteristics were associated with a risk factor for belonging to the second group, i.e., for not perceiving a clinically significant improvement after the BoNT/A treatment. The characteristics associated with an odds ratio (OR) significantly higher or lower than 1 (confidence level: 5%) were labeled as risk and protective factors, respectively.

## Figures and Tables

**Table 1:** Design of studies that assessed vestibulodynia treatment with injections of BoNT/A into the bulbocavernosus muscle.

Study	Sample Size	Placebo-Controlled	Randomized	Open Label	Dose/Side	Repeated Injections	Clinical Dimensions Monitored
Petersen et al., 2009 [23]	64	Yes	Yes	No	20 units	No	Day-to-day pain Sexual function Quality of life
Pelletier et al., 2016 [26]	19	No	No	Yes	50 units	No	Day-to-day pain Sexual function Quality of life
Diomande et al., 2019 [24]	31	Yes	Yes	No	50 or 100 units	Yes (6 months later)	Vulvar sensitivity Sexual function
Haraldson et al., 2020 [25]	88	Yes	Yes	No	20 units	No	PFM strength Sexual function

**Table 2:** Patients’ general, obstetric, gynecological and pain-related backgrounds at the beginning of the study.

Background	Characteristic	Mean ± SD	No. of Patients		%
General	Age (years)	41.9 ± 14.1			
	Body mass index (kg/m^2^)	22.4 ± 2.7			
	Academic background		Reading and writing	0/35	0.0
			Primary	1/35	2.9
			Secondary	6/35	17.1
			Undergraduate	10/35	28.6
			Graduate	18/35	51.4
			Postgraduate	0/35	0.0
	Work activity		Unemployed	2/35	5.7
			Pensioner	2/35	5.7
			Work incapacity	1/35	2.9
			Active worker	21/35	60.0
			Student	6/35	17.1
			Off work	1/35	2.9
			Housewife	2/35	5.7
	Smoke			8/35	22.9
	History of abuse			3/35	8.6
Obstetric	Pregnancies			16/35	45.7
	Vaginal deliveries			9/16	56.3
	Episiotomy			7/9	77.8
	Perineal tears			5/9	55.6
	Cesarean sections			6/16	37.5
Gynecological	Vulvovaginal trauma			7/35	20.0
	Vulvovaginal infections/inflammations			6/35	17.1
	Menopause (hormonal deficiencies)			12/35	34.3
Comorbidities	Cervical pain			15/35	42.9
	Lumbar pain			13/35	37.1
	Headaches			11/35	31.5
	Temporomandibular joint pain			13/35	37.1
	Allergies/intolerances			12/35	34.3
Vestibulodynia description	Inciting factor		Provoked	21/35	60.0
			Spontaneous	0/35	0.0
			Mixed	14/35	40.0
	Location		Generalized	27/35	77.1
			Localized	8/35	22.9
	Temporal pattern (provoked pain)		Punctual, short duration	26/35	74.3
			Intermit., few days	0/35	0.0
			Constant, few days	6/35	17.1
			Intermit., long time	1/35	2.9
			Constant, long time	1/35	2.9
	Temporal pattern (spontaneous pain)		Punctual, short duration	27/35	77.1
			Intermit., few days	1/35	2.9
			Constant, few days	1/35	2.9
			Intermit., long time	5/35	14.3
			Constant, long time	7/35	20.0
	Years since pain onset	6.9 ± 6.3			
Other symptoms	Myofascial pelvic pain			17/35	48.6

**Table 3:** Adverse events reported by patients after BoNT/A injections.

Adverse Event	Onset	Duration	Severity
Stress urinary incontinence	2 weeks after treatment	7 weeks	Mild
Difficulties in achieving climax	4 weeks after treatment	6 weeks	Mild

**Table 4:** Risk and protective factors against a non-clinically significant improvement after the BoNT/A treatment. OR: odds ratio. CI: confidence interval.

Factor	OR	95% CI	*p*-Value
Smoking	7.12	[1.10–46.20]	0.03
Associated painful syndromes (sensitization questionnaire)	6.88	[1.08–43.61]	0.03
Vestibular pain at the 3 o’clock position	0.15	[0.02–0.97]	0.04
Maximum VAS score (cotton swab test)	0.41	[0.17–0.96]	0.03
FSFI score (orgasm domain)	2.18	[1.00–4.78]	0.04

**Table 5:** Summary of activities performed in the visits.

	Previous Visit	Week 0	Week 8	Week 12	Week 24
Eligibility criteria	X				
Patient’s informed consent	X				
Medical history	X				
Physical examination - Genital trophism - Vestibular cotton swab test - Myofascial pelvic pain syndrome - Pudendal nerve assessment - Vulvar pain sensitivity (algometer)					
	X	X	X	X	X
	X	X	X	X	X
	X	X	X	X	X
	X	X	X	X	X
		X	X	X	X
Clinical questionnaires - VAS - FSFI - HADS - Marinoff’s Dyspareunia Scale - PGI-I					
		X	X	X	X
		X	X	X	X
		X	X	X	X
		X	X	X	X
			X	X	X
sEMG recording		X	X	X	X
Administration of BoNT/A		X			
Adverse events		X	X	X	X
Intercurrent medication		X	X	X	X
Morbidity		X	X	X	X

## Data Availability

The data are not publicly available since subjects enrolled in the study were not explicitly asked whether they consented to the sharing of their data.

## Supplementary Materials

The following supporting information can be downloaded at: https://www.mdpi.com/article/10.3390/toxins15080476/s1, Table S1. Mean ± standard deviation or frequency of occurrence of the categories (%) of the clinical questionnaires, physical ex-amination and RMS of sEMG signals at baseline (PV) and after BoNTA administration (W8, W12, W24), and *p*-values (*p*) obtained when comparing PV vs. {W8, W12, S24}.
