# Supplementary material for: Treatment of Vestibulodynia with Submucosal Injections of IncobotulinumtoxinA into Targeted Painful Points: An Open-Label Exploratory Study
Source: Toxins (Basel). 2023 Jul 25;15(8):476. doi: 10.3390/toxins15080476 (PMC10467121; doi:10.3390/toxins15080476)
Supplement: Supplementary file 1 [file toxins-15-00476-s001.zip › toxins-2451429-supplementary.pdf]

toxins

MDPI

Supplementary Materials: Treatment of Vestibulodynia with Submucosal Injections of Incobotulinumtoxina into Targeted Painful Points: An Open-Label Exploratory Study

Paula Villa-Muñoz, Monica Albaladejo-Belmonte, Francisco J. Nohales-Alfonso, Jose Alberola-Rubio and Javier Garcia-Casado

Table S1. Mean ± standard deviation or frequency of occurrence of the categories (%) of the clinical questionnaires, physical examination and RMS of sEMG signals at baseline (PV) and after BoNTA administration (W8, W12, W24), and *p*-values (*p*) obtained when comparing PV vs. {W8, W12, S24}.

Variable	PV	W8	*p*	W12	*p*	W24	*p*
VAS (day-to-day pain)							
Provoked	8.09 ± 1.46	5.30 ± 2.30	<0.001	5.20 ± 2.46	<0.001	5.20 ± 2.33	<0.001
Spontaneous	5.11 ± 2.22	4.07 ± 2.89	0.016	3.92 ± 3.40	0.076	4.92 ± 2.54	0.233
FSFI							
Desire	2.64 ± 1.25	2.96 ± 1.37	0.174	2.89 ± 1.34	0.169	2.98 ± 1.35	0.179
Arousal	2.64 ± 1.92	3.27 ± 1.85	0.022	3.07 ± 1.88	0.051	3.37 ± 1.62	0.019
Lubrication	2.93 ± 1.93	3.61 ± 1.97	0.035	3.20 ± 2.18	0.258	3.58 ± 1.68	0.134
Orgasm	2.52 ± 2.16	3.53 ± 2.05	0.002	3.17 ± 2.17	0.025	3.51 ± 1.85	0.004
Satisfaction	3.07 ± 1.92	3.92 ± 1.72	0.013	3.74 ± 1.76	0.023	3.61 ± 1.65	0.145
Pain	1.54 ± 1.25	2.80 ± 1.79	<0.001	2.40 ± 1.95	<0.001	3.21 ± 1.55	<0.001
Total	15.31 ± 8.78	20.10 ± 9.16	0.001	18.77 ± 9.95	0.006	20.26 ± 8.52	0.005
Marinoff's Dyspareunia scale							
No pain	3.13	6.25	0.012	6.67	0.019	10.00	0.045
Pain does not prevent intercourse	21.88	50.00		53.33		50.00	
Pain interrupts intercourse	40.63	34.38		30.00		26.67	
Pain prevents intercourse	34.38	9.38		10.00		13.33	
HADS							
Anxiety	7.20 ± 3.52	5.03 ± 3.56	<0.001	6.14 ± 4.31	0.004	4.74 ± 3.70	<0.001
Depression	4.66 ± 3.68	3.17 ± 3.08	0.005	3.26 ± 3.58	0.003	2.54 ± 2.75	0.003
Catastrophizing	19.69 ± 14.47	15.68 ± 10.65	0.039	14.62 ± 11.70	0.112	13.69 ± 12.29	0.041
PGI-I							
Very much better		11.42%		11.42%		9.38%	
Much better		34.29%		42.86%		37.50%	
A little better		34.29%		25.71%		28.13%	
No change		17.14%		20.00%		15.63%	
A little worse		2.86%		0.00%		3.13%	
Much worse		0.00%		0.00%		6.25%	
No. painful points (algometer)	5.12 ± 1.43	3.96 ± 2.40	0.038	4.22 ± 2.15	0.023	3.88 ± 2.25	0.004
Maximum VAS (algometer)	7.97 ± 1.34	5.59 ± 2.97	<0.001	5.73 ± 2.82	<0.001	5.86 ± 2.28	<0.001
PFMH, left side							
None	68.57%	66.67%	0.879	69.70%	0.160	56.67%	0.393
Mild	14.29%	15.15%		3.03%		6.67%	
Moderate	11.43%	9.09%		18.18%		26.67%	
Severe	5.71%	9.09%		9.09%		10.00%	
PFMH, right side							
None	68.57%	75.76	0.392	72.73%	0.251	76.67%	0.081
Mild	11.43%	6.06%		3.03%		0.00%	
Moderate	17.14%	6.06%		12.12%		13.33%	

Severe	2.86%	12.12%		12.12%		10.00%	
PFM tone, left side							
Normal	45.71%	60.00%	0.197	51.43%	0.593	60.00%	0.197
Hypertonic	54.29%	40.00%		48.57%		40.00%	
PFM tone, right side							
Normal	77.14%	77.14%	1.000	65.74%	0.157	68.57%	0.257
Hypertonic	22.86%	22.86%		34.29%		31.43%	
RMS (μV)							
Left side, contraction	3.91 ± 1.50	3.80 ± 1.52	0.681	3.83 ± 1.48	0.659	3.62 ± 1.52	0.183
Right side, contraction	4.00 ± 1.50	3.74 ± 1.45	0.259	3.56 ± 1.44	0.007	3.53 ± 1.56	0.208
Left side, relaxation	1.44 ± 0.42	1.53 ± 1.04	0.478	1.48 ± 0.91	0.293	1.32 ± 0.67	0.078
Right side, relaxation	1.54 ± 0.73	1.50 ± 1.04	0.092	1.32 ± 0.73	0.001	1.19 ± 0.49	0.100
